# Iron-regulated gene *ireA* in avian pathogenic *Escherichia coli* participates in adhesion and stress-resistance

**DOI:** 10.1186/s12917-016-0800-y

**Published:** 2016-08-17

**Authors:** Yaxin Li, Jianjun Dai, Xiangkai Zhuge, Haojin Wang, Lin Hu, Jianluan Ren, Ling Chen, Dezhi Li, Fang Tang

**Affiliations:** Key Laboratory Animal Bacteriology, Ministry of Agriculture, College of veterinary medicine, Nanjing Agricultural University, No.1 Weigang, Nanjing, 210095 Jiangsu province People’s Republic of China

**Keywords:** APEC, *IreA*, Virulence, Adhesion, Stress resistance

## Abstract

**Background:**

Avian pathogenic *Escherichia coli* (APEC) causes avian colibacillosis, which results in economic and welfare costs in the poultry industry worldwide. The pathogenesis of avian pathogenic *E. coli* strains is not well defined. Here, the function of an outer membrane protein encoded by the *ireA* gene of avian pathogenic *E. coli* strain DE205B was investigated.

**Results:**

The *ireA* gene was distributed in 32.9 % (46/140) of tested *E. coli* strains, with high percentages in the phylogenetic ECOR groups B2 (58.8 %, 10/17) and D (55.9 %, 19/34). The gene expression level of *ireA* of APEC strain DE205B in high Fe M9 media was 1.8 times higher (*P* < 0.05) than that in low Fe M9 media. An *ireA* deletion mutant and complementary strain were constructed. Compared with the wild-type strain DE205B, the expression of most ferric uptake genes in the *ireA* deletion mutant were significantly upregulated (*P* < 0.05). The adhesion ability of the *ireA* deletion mutant to DF-1 cells was significantly decreased. The survival rate of *ireA* deletion mutant was reduced 21.17 % (*P* < 0.01), 25.42 (*P* < 0.05) and 70.0 % (*P* < 0.01) under alkali, high osmolarity, and low temperature (4 °C) conditions, respectively, compared with the wild-type strain.

**Conclusions:**

The results suggested that the protein encoded by the iron-regulated gene *ireA* has roles in adhesion and stress resistance in avian pathogenic *E. coli*.

**Electronic supplementary material:**

The online version of this article (doi:10.1186/s12917-016-0800-y) contains supplementary material, which is available to authorized users.

## Background

Avian pathogenic *Escherichia coli* (APEC), a subgroup of extra-intestinal pathogenic *E. coli* (ExPEC) causes avian colibacillosis and imposes economic losses on the poultry industry worldwide [1]. However, the pathogenesis of APEC is poorly understood. Many virulence genes have been studied to identify virulence factors in APEC, including those involved in adhesion, iron-regulation, toxin/cytotoxin production and serum resistance [2]. Iron is an essential element involved in important biological processes [3]. Biological activities in cells, such as peroxide reduction, nucleotide biosynthesis and electron transport, are facilitated by iron ions [4]. Extra-intestinal sites have low iron contents; therefore, ExPEC strains struggle to take up iron from the host during infection [5]. During natural infection, the initiation, progression and transmission of most bacterial infections depend on the ability of the invading pathogen to acquire iron from the complicated environment [6]. During iron acquisition, the cell must produce transmembrane receptors for siderophores that chelate iron ions [7]. There are various receptors that chelate iron ions encoded by bacterial genes, such as *chuA*, the *SitABCD* system, *iron*, *iha*, *iutA*, and *ireA*. Outer membrane protein ChuA participates in heme acquisition in enterohemorrhagic *E. coli* and uropathogenic *E. coli* (UPEC) strains, and is important for the pathogenicity of APEC [8, 9]. The *SitABCD* system, identified in the APEC strain MT512 by comparative genomic analysis, was reported to be associated with the pathogenicity of APEC [9, 10].

*IreA* was suggested to be involved in Fe acquisition and to act as an iron-regulated virulence gene in the blood- or urine-derived ExPEC *E. coli* isolated from humans [11]; however, its exact role in APEC strains remains unknown. Herein, an *ireA* deletion mutant was constructed to study the *ireA* gene function in the APEC strain DE205B.

## Results

### Prevalence of the *ireA* gene among *E. coli* Strains

As shown in Table 1, *ireA* was present in 32.9 % (46/140) of *E. coli* strains, with 19.0 % (12/63) in phylogenetic ECOR group A, 19.2 % (5/26) in B1, 58.8 % (10/17) in B2 and 55.9 % (19/34) in group D (Additional file 1: Table S1). Thus, the *ireA* gene was significantly more frequently distributed in the B2 and D groups than in the A and B1 groups.

**Table 1:** Distribution of the *ireA* gene in *Escherichia coli* strains

ECOR group	Strain counts	Strain counts positive for *ireA*	Percentage
A	63	12	19.0 %
B1	26	5	19.2 %
B2	17	10	58.8 %
D	34	19	55.9 %
Total	140	46	32.9 %

### Expression of the *ireA* gene

**Fig. 1:**
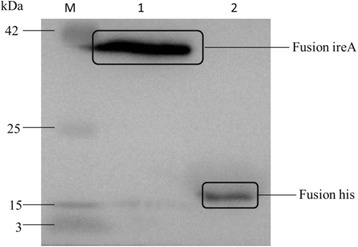
Expression of *ireA* by western blotting. Expression of fusion *ireA* protein was detected by immunoblotting. Fusion *ireA* was detected in pET32a(+), while there is only his protein was detected in the blank plasmid. Lane M, protein marker; lane 1, fusion *ireA*; lane 2, fusion his

The *ireA* gene expression was tested by Immunoblotting. Western blotting was performed with anti-His serum, showing expected fusion protein bands for *ireA* (39 kDa) from strains DE205B. However, only fusion his protein (18 kDa) was detected from the blank plasmid control (Fig. 1). These results indicated that *ireA* was expressed under laboratory conditions.

### Gene expression of *ireA* in M9 media with different iron content

**Fig. 2:**
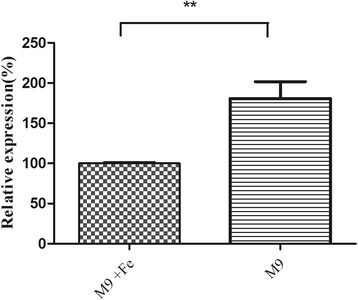
Expression of *ireA* in M9 media. The first and second columns represent the relative gene expressions of *ireA* in M9 media with low and high Fe content, respectively. The gene expression levels showed significant differences in the two kinds of media (*P* < 0.01)

The relative gene expression of *ireA* in low Fe M9 media was 1.8 times higher than that in high Fe M9 media (*P* < 0.01) (Fig. 2).

### Growth curve

**Fig. 3:**
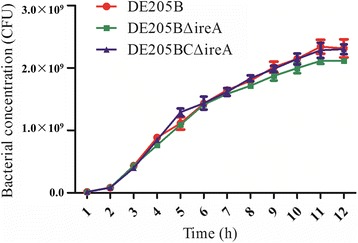
Growth curve of different strains. Growth curves of the wild-type strain DE205B, mutant strain DE205BΔ*ireA* and complementary strain DE205BCΔ*ireA*. Bacterial growth was estimated by plate counting as Colony Forming Units (CFU)

The CFU of the wild-type strain DE205B, mutant strain DE205BΔ*ireA* and the complementary strain DE205BCΔ*ireA* was monitored for 12 h. There was no significant difference between the growth curves of the wild-type and mutant strains (Fig. 3), which indicated that deletion of *ireA* had no effects on the growth of DE205B.

### Expression variations of Ferric uptake system and adherence genes

**Fig. 4:**
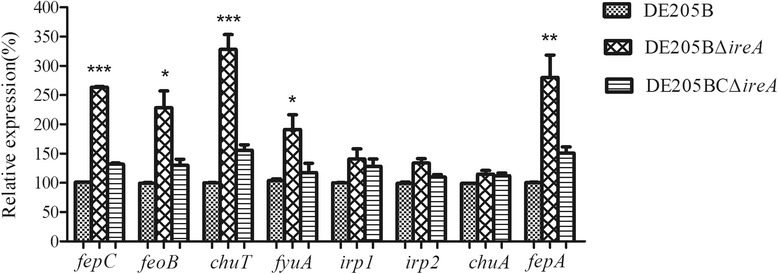
Expressions of Fe acquisition genes. Expressions of Fe acquisition genes of the wild-type strain DE205B, mutant strain DE205BΔ*ireA* and complementary strain DE205BCΔ*ireA* were tested by qPCR. The relative expression levels in the different mutant strains were calculated using the 2^-△△Ct^ method

The expressions of *fepC*, *feoB*, *chuT*, *fyuA*, *irp1*, *irp2*, *chuA* and f*epA* were detected in the mutant and compared with their expressions in the wild-type strain DE205B. Most of the ferric uptake genes in the mutant strain DE205BΔ*ireA*, except *irp1*, *irp2* and *chuA*, were significantly upregulated (*P* < 0.05) (Fig. 4). *Irp1*, *irp2* and *chuA* were non-significantly upregulated. Furthermore, the expressions of the eight ferric uptake genes in the complementary strain DE205BCΔ*ireA* were restored to wild-type levels.

**Fig. 5:**
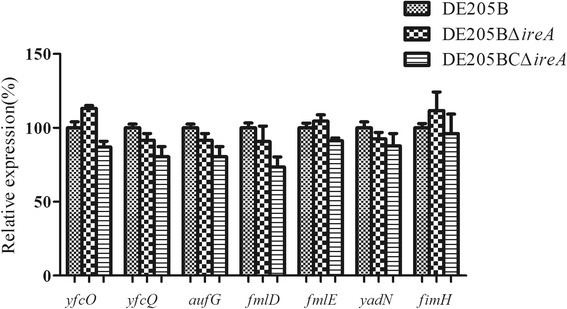
Expressions of adhesion genes. Expressions of adhesion genes, including *yfcO*, *yfcQ*, *aufG*, *fmlD*, *fmlE*, *yadN* and *fimH*, of the wild-type strain DE205B, mutant strain DE205BΔ*ireA* and complementary strain DE205BCΔ*ireA* were tested by qPCR. The relative expression levels in the different mutant strains were calculated using the 2^-△△Ct^ method

The expression of adherence genes *yfcO*, *yfcQ*, *aufG*, *fmlD*, *fmlE*, *yadN* and *fimH* showed no significant difference between the wild-type strain DE205B and the *ireA* mutant strain (Fig. 5).

### Adherence assays

**Fig. 6:**
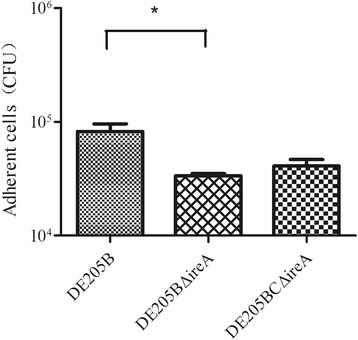
Adherence assays. The adhesion abilities of the wild-type strain DE205B, mutant strain DE205BΔ*ireA* and complementary strain DE205BCΔ*ireA* to DF-1 cells were tested. The numbers of adherent cells were calculated by plate counting. The number of DE205BΔ*ireA* mutant bacteria adhering to DF-1 cells was significant decreased compared with the number of DE205B wild-type bacteria (*P* < 0.05)

The number of mutant DE205BΔ*ireA* bacteria adhering to DF-1 cells was significantly decreased compared with the number of DE205B wild-type adherent cells (*P* < 0.05) (Fig. 6). The adhesion to DF-1 cells of complementary strain DE205BCΔ*ireA* was partly restored to wild-type levels.

### Animal infection

The LD_50_ of the wild-type, deletion mutant and complementary strains were 1.74 × 10^5^, 2.45 × 10^5^, and 3.16 × 10^5^, respectively (Table 2). The results showed that there was no significant difference between the LD_50_ of the wild-type and deletion mutant strains, indicating that the deletion of *ireA* gene has no obvious effect on the virulence of DE205B toward ducks.

**Table 2:** LD_50_ of wild-type and mutant strains

Challenge dose (CFU/ml)	Duck mortality		
	DE205B	DE205BΔ*ireA*	DE205BCΔ*ireA*
10^8^	7/7	7/7	7/7
10^7^	7/7	7/7	7/7
10^6^	6/7	6/7	6/7
10^5^	3/7	2/7	1/7
LD_50_	1.74 × 10^5^	2.45 × 10^5^	3.16 × 10^5^

### Determination of resistance to environmental stress

**Fig. 7:**
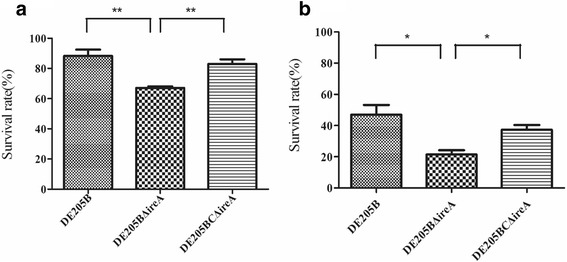
Resistance to alkaline and high osmolarity. The resistance to alkali (**a**) and high osmolarity (**b**) of wild-type strain DE205B, mutant strain DE205BΔ*ireA* and complementary strain DE205BCΔ*ireA* was tested by exposing the strains to alkaline and high osmolarity conditions and calculating their survival rates. Compared with the wild-type strain, the survival rate of the *ireA* deletion mutant was reduced 21.17 % (*P* < 0.01) and 25.42 (*P* < 0.05) under alkaline and high osmolarity conditions, respectively

**Fig. 8:**
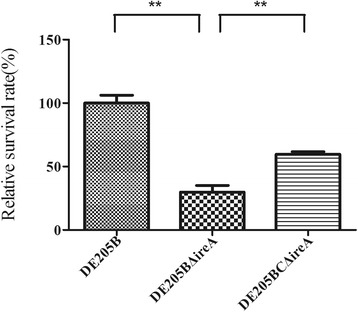
Challenge under the low temperature of different strains. Each bacterial suspension with concentration of 10^7^ CFU/ml (DE205B, DE205BΔ*ireA* or DE205BCΔ*ireA*) were incubated at 4 °C for 7days. The survival rates of wild-type and mutant strains were calculated by plate counting and compared using GraphPad Prism 5. The *ireA* deletion mutant was reduced 70.0 % (*P* < 0.01) under the low (4 °C) temperature compared to that of the wild type strain

Compared with the wild-type strain, the survival rate of the *ireA* deletion mutant was reduced by 21.17 % (*P* < 0.01) and 25.42 % (*P* < 0.05) under alkali and high osmolarity conditions, respectively (Fig. 7). The resistance to alkali and high osmolarity conditions of the complementary strain DE205BCΔ*ireA* was almost restored to wild-type levels. For the temperature challenge, compared to the wild type strain, the *ireA* deletion mutant was reduced 70.0 % (*P* < 0.01) under the low (4 °C) temperature (Fig. 8). The resistance to low temperature of the complementary strain DE205BCΔ*ireA*was partly restored. The results showed that presence of the *ireA* gene increased the stress-resistance of APEC strain DE205B.

## Discussion

The *ireA* gene is an iron-regulated gene and is involved in iron acquisition in human pathogenic *E. coli* isolates and our study proved this protein functions in APEC. Additionally, we identified two new functions of *ireA* using the deletion mutant. In the present study, *ireA* was demonstrated to contribute to the adhesion to DF-1 cells. Moreover, the expressions of several adhesion genes were tested and the results showed no significant differences between wild-type and mutant strain, indicating that the *ireA* gene indeed plays a role in adhesion. Tarr et al., identified adhesin Iha from an O157:H7 strain of *E. coli* [12]. This adhesin shared high similarities with several identified or putative siderophore receptors. Siderophore receptor IrgA was reported to contribute to growth in the rabbit ileal loop model *in vivo* and to enhance virulence in an infant mouse model, suggesting a possible role in colonization [13, 14]. Here, we proved that iron-regulated gene *ireA* plays a role in the adhesion of APEC strains. The *ireA* gene also increased stress-resistance under alkali and high osmolarity conditions, as well as underlow temperature. Thus, the redundancy of siderophore receptors might reflect their multifunctional roles.

*E. coli* strains were reported to be classified into four main phylogenetic groups (A, B1, B2, and D) [15]. Virulent ExPEC strains mainly belong to phylogroup B2 and D, whereas most commensal strains belong to phylogroup A [16]. The *ireA* gene was distributed more frequently in the B2 (58.8 %) and D (55.9 %) groups than in the A (19 %) and B1 (19.2 %) groups, indicating that *ireA* might be associated with the virulence of the APEC strain DE205B. Thus, the result correlated with that Russo’s report: *ireA* was detected in 13 (26 %) of 50 random clinical isolates from patients and in none (0 %) of 14 fecal isolates, which presumably represented commensal strains [11]. Taken together, these results indicated that *ireA* might be a virulence gene in both human and avian ExPEC *E. coli* strains.

The expression level of *ireA* in DE205B was decreased in high Fe M9 media compared with that in low Fe media, indicating that *ireA* is involved in iron-regulation in this APEC strain. This result agreed with the report of Russo et al., who found that *ireA* was a iron-regulated virulence gene in the blood- or urine-derived isolates of ExPEC *E. coli* [11]. Most ferric uptake genes, such as *fepC*, *feoB*, *chuT*, *fyuA* and f*epA* were upregulated in the *ireA* deletion mutant strain. This might represent a compensatory function for *ireA* gene deletion. Fe acquisition is important for many microorganisms, especially for pathogens that grow in the host, which attempts to limit Fe availability. It is thought that pathogens harbor multiple Fe acquisition systems to ensure that Fe is gained from the host cells to provide a selective advantage. Alternatively, certain siderophores, and their cognate receptors, might be more active in certain environments, such as inside or outside the gastrointestinal tract [17, 18]. Moreover, multiple systems might represent ‘alternatives’ that protect against the disruption of one system caused by genomic rearrangements or mutations.

In the duck infection experiments, the LD_50_ showed no significant difference between the wild-type DE205B and the *ireA* deletion mutant strain. Thus, it seemed that *ireA* deletion had no obvious effect on the virulence of DE205B. However, DE205B has several Fe acquisition systems. We showed that most of the other Fe acquisition genes were upregulated in the *ireA* gene deletion mutant. Thus, the *ireA* gene might indeed contribute to the virulence of the APEC strain DE205B, while other Fe acquisition genes displayed compensatory functions when the *ireA* gene was deleted.

## Conclusions

In summary, the *ireA* gene was mainly distributed in the more virulent phylogenetic ECOR group B and D. Compared with the wild-type strain, the adhesion and resistance to environmental stress of the *ireA* deletion mutant were significant decreased. This indicated that *ireA* is a Fe iron-regulated gene that aids adhesion and stress-resistance in the APEC strain DE205B.

## Methods

### Bacterial strains, plasmids and growth conditions

The APEC strain DE205B, which was characterized previously, was isolated from the brain of a duck with neurological symptoms in our previous work [19–22]. Its serotype is O2:K1, which is one of the predominant serotypes of APEC [9, 23]. All *E. coli* strains were grown in Luria-Bertani (LB) medium at 37 °C with shaking or on LB plates with 2 % agar. When necessary, the LB medium was supplemented with appropriate antibiotics: ampicillin (Amp; 100 μg/mL) and kanamycin (Kan; 50 μg/mL), unless otherwise specified. Information on the main strains and plasmids used in this study are listed in Table 3. M9 minimal medium was used to assess the expression of *ireA*, which was prepared as follows: an aliquot was mixed with200 ml of 5 × M9 salt solution (containingNa_2_PO_4_ · 7 H_2_O, 12.8 g; KH_2_PO_4,_ 3.0 g; NaCl, 0.5 g; NH_4_Cl, 1.0 g;),2 ml of 1 M MgSO_4_, 20 ml of 20 % glucose solution, 0.1 ml of1 M CaCl_2_, and then dissolved in1000 ml double distilled water andfiltrated through a 0.22-μm membrane.

**Table 3:** Bacterial strains and plasmids used in the present study

Strain or plasmid	Characteristics	Reference
Strain		
DE205B	O2:K1, NalR	[16, 19]
DE205BΔ*ireA*	*ireA* deletion mutant strain	This study
DE205BCΔ*ireA*	*ireA* complementary strain	This study
Plasmid		
pKD46	Amp, express λ red recombinase	[29]
pKD4	kan, template plasmid	[29]
pSTV28	Cm, expression using lac promotor	TAKARA
pCP20	Cm, Amp, yeast Flp recombinase gene, FLP	[29]

### Prevalence of *ireA* among *E. coli* Strains

The distribution of *ireA* among 140 *E. coli* strains (Additional file 1: Table S1) maintained in our laboratory was tested using PCR. The PCR primers were designed using Primer Premier 5. The specific primers *ireA*-F and *ireA*-R are listed in Table 4. The PCR conditions were: 5 min at 95 °C for initial denaturing; 30 cycles of 1 min at 95 °C, 30 s at 50 °C and 1 min at 72 °C; followed by 10 min at 72 °C for extension. The DNA of DE205B and ddH_2_O were used as positive and negative templates, respectively. The PCR products were electrophoresed through 1 % agarose gels. Phylogenetic *E. coli* reference (ECOR) groups of single isolates of the 140 *E. coli* strains were determined using triplex PCR, as described previously [16, 24].

**Table 4:** Primers used in the present study

Primers	Primer sequence (5′–3′)	Gene	Reference
ireA-F	AGATACGCTTGTTGTTAC	*ireA*	This study
ireA-R	CATTCCATGCTGCTAC		
ireAMu-F	GTAAATTCCCTCTTTGCTAACGCAAATCATTATCATTACGCCTTTGGCAAAGGGAAATTTGTGTAGGCTGGAGCTGCTTCGA	Upstream region of *ireA*	This study
ireAMu-R	CATTACTGATTACTACACTGGTACCTGAGGCTCACGGCCTCAGGTTGTCTTTATATACTCCATATGAATATCCTCCTTAG	Downstream region of *ireA*	
ireA-1	TACTCCCCATCCCCGGGCAA	*ireA*	This study
ireA-2	GGGGGCAGCAATATCCGGTG		
k1	CAGTCATAGCCGAATAGCCT	*kanR*	[27]
k2	CGGTGCCCTGAATGAACTGC		
ireACo-F	TACGGATCCCTGAACAATAGCGAT	*ireA*	This study
ireACo-R	GCTAAGCTTTTACAACCTGGAAAC		
QireA-F	AAACATGGGATGGCGTACTT	*ireA*	This study
QireA-R	AATCAATGGGCCTGACAGATAG		
QFeoB-F	GCACTCTTTGTGCATGGTATTC	*FeoB*	This study
QFeoB-R	TGGCAGCACGGTGTTAAT		
QfepA-F	CAATGCGCCAGAACATAAAGAG	*fepA*	This study
QfepA-R	TGTCGAGGTTGCCATACAAG		
QfepC-F	TCGTTACGCCAGCCATTT	*fepC*	This study
QfepC-R	TGCAGCGCAGACCATAAA		
QfyuA-F	ATGCCTATGTGGGATGGAATG	*fyuA*	This study
QfyuA-R	CCAGTCATCGGTGGTGTATTT		
Qirp1-F	GGCGAACCCTGCTATGTATT	*irp1*	This study
Qirp1-R	GTCCATGCAGTACCAGCTAAA		
Qirp2-F	GCGGCTGATTACCAACAATTAC	*irp2*	This study
Qirp2-R	CTGGATCAGGTTGCTCTCTTC		
QChuA-F	TAGGCCACATCAAGGCTAAAC	*ChuA*	This study
QChuA-R	CGGCGACAACTATGTCGTATAA		
QdnaE-F	ATGTCGGAGGCGTAAGGCT	*dnaE*	This study
QdnaE-R	TCCAGGGCGTCAGTAAACAA		
P1	TTGCCGGAATTCTCAGGGGGCCA GTCTACTGAATGAG	fusion *ireA*	This study
P2	ACCGTCGTCGACTTAGTGATGATGATGAT GATGAGAACCATACAGGGAAGACATAGGG		
QyfcO-F	TGCATAACTCGTCATGTCCTGTCTCCGTTG	*yfcO*	This study
QyfcO-R	AGCGTTTTCTCAATCGTTCCGCCCGTC		
QyfcQ-F	CCTGCATTTCTACGGCAATTTAC	*yfcQ*	This study
QyfcQ-R	CGGTCACCATCAGGTCTTTAC		
QaufG-F	TGCTCTGGCAACATCAGTAG	*aufG*	This study
QaufG-R	ACTAACAGGTAAAGTCAGGAAAGT		
QfmlD-F	CAGGCTCCCTACAGTCATATAAAG	*fmlD*	This study
QfmlD-R	TCGGTGTCTTATCACCAATATCC		
QfmlE-F	CAGTGCCAGACAATTTCCAAC	*fmlE*	This study
QfmlE-R	CCGTTAAATGCAACCTGAACTC		
QyadN-F	CCACTGTTAATGGCGGTGTA	*yadN*	This study
QyadN-R	TTTAGCCAGGCGAGAAGAAC		
QfimH-F	CTTATGGCGGCGTGTTATCT	*fimH*	This study
QfimH-R	CGGCTTATCCGTTCTCGAATTA		

### Expression of the *ireA* gene

The expression of the *ireA* gene was tested by fusion expression and western blotting as previously reported [25, 26]. The fusion fragment of *ireA* (including the *ireA* promoter and 579bp of *ireA* sequence) and His tag were inserted into plasmid pET32a(+). The fusion PCR primers P1 and P2 were listed in Table 4. Plasmid pET32a(+) without the fusion fragment was used as a blank control. For immunoblotting, protein samples were subjected to sodium dodecylsulfate polyacrylamide gel electrophoresis (SDS-PAGE) and transferred to polyvinylidene fluoride membranes (Amersham Pharmacia Biotech, Piscataway, NJ, USA), as described previously [27]. Anti-His serum was the primary antibody, horseradish peroxidase-conjugated goat anti-mouse IgG was the secondary antibody and 3,3′-diaminobenzidine was used as the substrate.

### Regulation of *ireA* expression in M9 media

M9 minimal medium was used to assess the expression of *ireA*.M9 medium or M9 medium with Fe (0.1 mM Fe(NO_3_)_3_) were used as low and high Fe content media, respectively. DE205B was cultured in both M9 media to the mid-log phase and the expression of *ireA* was detected by quantitative real-time reverse transcription PCR(qRT-PCR). The real –time PCR primers were designed by PrimerQuest Tool IDT (http://sg.idtdna.com/primerquest/Home/Index). Briefly, total RNA was extracted from 1 ml of bacteria culture using the Trizol RNA isolation protocol (Invitrogen, Shanghai, China) and cDNA was amplified by reverse transcription according to the instructions of the primeScript RT reagent Kit (Takara). Quantitative real-time PCR (qPCR) was carried out using the ABI Prism 7300 and Sequence Detection System software version 1.4 (Applied Biosystems, Foster City, CA, USA), according to the instructions of the SYBR Premix Ex Taq (Takara, Dalian, China). QPCR primers for *ireA* (Q*ireA*-F and Q*ireA*-R) are listed in Table 4. *DnaE* was used as a reference gene. Assays were performed three times. The relative expressions of *ireA* in different media were calculated using the 2^-△△Ct^ method [28]. Statistical analysis was performed using an unpaired *t* test in Graphpad Prism 5.0.

### Construction of the *ireA* deletion mutant and complementary strain

An *ireA* knockout strain of DE205B was constructed using the lambda red recombinase system described by Datsenko and Wanner [29]. The specific primers *ireA*Mu-F and *ireA*Mu-R were designed to amplify the target gene *ireA*. The kanamycin resistance gene, which contained sequences homologous to the 5′ and 3′ ends of the target sequence, was amplified using plasmid pKD4 as a template. The PCR products were then transformed by electroporation into DE205B containing the lambda red recombinase expression plasmid pKD46. The transformed bacterial cells were first incubated at 30 °C for 2 h in super optimal broth with catabolite repression (SOC) broth, and then grown on LB agar containing kanamycin at 37 °C. Mutants were confirmed by PCR and sequenced using primers k1 and k2 (Table 4) in combination with primers *ireA*-1 and *ireA*-2 (Table 4) flanking the *ireA* region. To remove the kanamycin resistance gene, plasmid pCP20 was transformed into the mutant and a kanamycin sensitive mutant strain was selected. Finally, the *ireA* deletion mutant strain without kanamycin resistance was named as DE205Bδ*ireA* (Additional file 2: Figure S1).

To construct the complementary strain, the *ireA* gene, including its putative promoters, was amplified using primers *ireA*Co-F and *ireA*Co-R (Table 4). The following amplification program were used: 5 min at 95 °C for initial denaturing; 35 cycles of 30 s at 95 °C, 30 s at 55 °C and 2.5 min at 72 °C; 10 min at 72 °C for extension. The PCR product of *ireA* gene was purified and subcloned into plasmid pSTV-28. The complementary strain DE205BCΔ*ireA* was generated by transforming vector pSTV-28-*ireA *into the deletion mutant (Additional file 3: Figure S2).

### Growth curve

The growths of the DE205B wild-type and mutant strains were compared in LB medium at 37 °C over a course of 12 h, starting at 10^7^ CFU/ml. Bacterial growth was estimated by plate counting as Colony Forming Units (CFU). Assays were performed three times.

### The expression of the Ferric uptake system and adherence genes

The effect of *ireA* deletion on the regulation of the ferric uptake system, including *fepC*, *feoB*, *chuT*, *fyuA*, *irp1*, *irp2*, *chuA* and *fepA* was detected using qRT- PCR. Seveal adherence genes, including *yfcO*, *yfcQ*, *aufG*, *fmlD*, *fmlE*, *yadN* and *fimH* were also selected to test their expression levels. The wild-type DE205B and the *ireA* deletion mutant were cultured in LB to mid-log phase and total RNA was extracted from 1 ml of bacterial culture using the Trizol RNA isolation protocol (Gibco BRL, USA, cat. no.15596-026). cDNA was reverse transcribed and real-time PCR was carried out as described above. The qRT-PCR primers for the ferric uptake system and adherence genes are listed in Table 4. The real-time PCR primers were designed unsing PrimerQuest Tool IDT (http://sg.idtdna.com/primerquest/Home/Index). The relative expression levels of the genes were calculated using the 2^-△△Ct^ method [28]. Assays were performed three times, and the statistical analysis was performed using an unpaired *t* test in Graphpad Prism 5.0. On the figures, error bars indicate the standard deviation.

### Adherence assays

The adherence assay was performed as described previously [24]. Briefly, chicken embryo fibroblast (CEF) DF-1 cells were seeded at approximately 1 × 10^5^ cells per well in 24-well tissue culture trays (TPP, Shanghai, China) and grown in Dulbecco’s modified Eagle medium (DMEM) with 10 % fetal bovine serum at 37 °C in a 5 % CO_2_ humidified atmosphere without antibiotics. DF-1 cells were washed once with DMEM and then inoculated with 500 ul of 2 × 10^7^ CFU/ml bacteria per well for 2 h at 37 °C in the presence of 5 % CO_2_. The cells were washed, lysed with ddH_2_O, and the number of bacterial cells was calculated by plate counting. In all assays, wells only containing DF-1 cells were used as negative controls. The adherence assays were conducted three times. The statistical analysis was performed using an unpaired *t* test in Graphpad Prism 5.0.

### Animal infections

Animal infections were performed as described previously [19, 20]. We purchased 7-day-old ducklings and young duck feeds from Anhui Poultry Farm (Anhui, China). Bacterial strains were cultured to the exponential phase, harvested, washed three times in PBS and then adjusted to the appropriate doses. Twenty-five 7-day-old ducks were inoculated intramuscularly 0.2 ml of each bacterial suspension (DE205B, DE205BΔ*ireA* or DE205BCΔ*ireA*) at four concentrations (5 × 10^8^ CFU/ml, 5 × 10^7^ CFU/ml, 5 × 10^6^ CFU/ml, and 5 × 10^5^ CFU/ml). Assays were performed three times. Seven ducks were used for each dose. Seven ducks were injected with PBS as a negative control. Death of the ducks was monitored for 7 days post infection. We calculated the LD_50_ of each strain using the method described by Spearman-Karber [30].

### Determination of resistance to environmental stress

Resistance to environmental stress was tested for the wild-type and the mutant strain, as described by La Ragione et al. [31]. Bacteria were cultured in LB broth overnight and harvested by centrifugation. The cells were resuspended in PBS and adjusted to 10^7^ CFU/ml in PBS. For alkali challenge, 100 μl of adjusted cells were mixed with 100 μl Tris buffer (1 M, pH10.0) and 800 μl ddH_2_O (final concentration, 100 mM, pH10.0) and incubated at 37 °C for 30 min. For high osmolarity endurance, cells were mixed with an equal volume of 4.8 M NaCl (final concentration, 2.4 M) and incubated at 37 °C for 1 h. Bacteria were exposed to PBS (pH 7.0) as a control.

Temperature challenge was performed as previously described with modifications [32], each bacterial suspension, at a concentration of 10^7^ CFU/ml (DE205B, DE205BΔ*ireA* or DE205BCΔ*ireA*) was incubated at 4 °C for 7days. Assays were performed three times. The survival rates of wild-type and mutant strains were calculated by plate counting and compared using GraphPad Prism 5.

## Additional files

Additional file 1: Table S1. Detailed information about the distribution of the *ireA* gene in APEC strains. (DOCX 21 kb) Additional file 2: Figure S1. Technical routes of construction of the *ireA* deletion mutant. (TIF 98 kb) Additional file 3: Figure S2. Technical routes of construction of the *ireA* complementary strain. (TIF 102 kb)
