# Supplementary material for: Iron-regulated gene ireA in avian pathogenic Escherichia coli participates in adhesion and stress-resistance
Source: BMC Vet Res. 2016 Aug 17;12:167. doi: 10.1186/s12917-016-0800-y (PMC4988017; doi:10.1186/s12917-016-0800-y)
Supplement: Additional file 1: Table S1. — Detailed information about the distribution of the ireA gene in APEC strains. (DOCX 21 kb) [file 12917_2016_800_MOESM1_ESM.docx]

**Supplementary Table 1.** **Detailed information about distribution of the *ireA* gene in APEC strains**

| No. | strains | source | location | ECOR group | ireA presence |
| --- | --- | --- | --- | --- | --- |
| 1 | DE205B | duck | Anhui | B2 | + |
| 2 | DE183 | duck | Anhui | B2 | + |
| 3 | DE164 | duck | Anhui | B2 | + |
| 4 | DE452 | duck | Anhui | D | + |
| 5 | DE209 | duck | Jiangsu | D | + |
| 6 | DE125 | duck | Anhui | D | + |
| 7 | DE162 | duck | Anhui | D | + |
| 8 | DE126 | duck | Anhui | B2 | + |
| 9 | DE134 | duck | Shandong | D | + |
| 10 | DE158 | duck | Anhui | B2 | + |
| 11 | DE182 | duck | Anhui | B2 | + |
| 12 | DE359 | duck | Jiangsu | B2 | + |
| 13 | DE130 | duck | Anhui | D | + |
| 14 | DE135 | duck | Shandong | D | + |
| 15 | DE165 | duck | Jiangsu | D | + |
| 16 | DE207 | duck | Jiangsu | B2 | + |
| 17 | DE065B | duck | Jiangsu | A | + |
| 18 | DE124 | duck | Anhui | B1 | + |
| 19 | DE007B | duck | Jiangsu | A | + |
| 20 | DE046B | duck | Anhui | D | + |
| 21 | DE013B | duck | Jiangsu | A | + |
| 22 | DE170 | duck | Jiangsu | D | + |
| 23 | DE178 | duck | Jiangsu | A | + |
| 24 | DE117 | duck | Jiangsu | A | + |
| 25 | DE193 | duck | Anhui | D | + |
| 26 | DE434 | duck | Anhui | A | + |
| 27 | DE131 | duck | Anhui | D | + |
| 28 | DE012 | duck | Jiangsu | D | + |
| 29 | DE123 | duck | Jiangsu | A | + |
| 30 | DE035 | duck | Anhui | D | + |
| 31 | DE175 | duck | Jiangsu | D | + |
| 32 | DE462 | duck | Anhui | D | + |
| 33 | DE115 | duck | Jiangsu | A | + |
| 34 | DE112 | duck | Jiangsu | A | + |
| 35 | DE194 | duck | Anhui | D | + |
| 36 | E058 | duck | Jiangsu | B1 | + |
| 37 | CVCC251 | chicken | Guangdong | B2 | + |
| 38 | CVCC1502 | piglet | Shanghai | B2 | + |
| 39 | CVCC193 | piglet | Beijing | A | + |
| 40 | CVCC230 | piglet | Shanghai | B1 | + |
| 41 | CVCC1522 | piglet | Shanghai | B1 | + |
| 42 | CVCC1526 | piglet | Zhejiang | A | + |
| 43 | CVCC1493 | piglet | Beijing | B1 | + |
| 44 | CVCC241 | calve | Sichuan | D | + |
| 45 | CVCC221 | calve | France | D | + |
| 46 | CVCC238 | calve | Beijing | A | + |
| 47 | DE127 | duck | Jiangsu | B2 | - |
| 48 | DE128 | duck | Anhui | D | - |
| 49 | DE142 | duck | Shandong | B2 | - |
| 50 | DE017 | duck | Jiangsu | B2 | - |
| 51 | DE95 | duck | Jiangsu | A | - |
| 52 | DE20 | duck | Anhui | B1 | - |
| 53 | CVCC236 | calve | Beijing | A | - |
| 54 | CVCC1506 | piglet | Jilin | A | - |
| 55 | CVCC1521 | piglet | Shanghai | D | - |
| 56 | CVCC1491 | piglet | Beijing | B1 | - |
| 57 | CVCC1503 | piglet | Beijing | A | - |
| 58 | CVCC1494 | piglet | Beijing | D | - |
| 59 | CVCC1546 | calve | Sichuan | B1 | - |
| 60 | CVCC1539 | yak | Gansu | D | - |
| 61 | CVCC1498 | piglet | Guangxi | B2 | - |
| 62 | CVCC1497 | piglet | Beijing | A | - |
| 63 | CVCC1513 | piglet | Beijing | A | - |
| 64 | CVCC1514 | piglet | Beijing | A | - |
| 65 | CVCC219 | calve | Britain | B2 | - |
| 66 | DE204 | duck | Anhui | A | - |
| 67 | DE460 | duck | Anhui | D | - |
| 68 | DE202 | duck | Jiangsu | B1 | - |
| 69 | DE299 | duck | Jiangsu | A | - |
| 70 | DE196 | duck | Anhui | B1 | - |
| 71 | DE345 | duck | Jiangsu | B2 | - |
| 72 | DE416 | duck | Jiangsu | A | - |
| 73 | DE089 | duck | Jiangsu | A | - |
| 74 | DE368 | duck | Jiangsu | A | - |
| 75 | DE070B | duck | Jiangsu | B1 | - |
| 76 | DE099 | duck | Jiangsu | A | - |
| 77 | DE059 | duck | Jiangsu | A | - |
| 78 | DE129 | duck | Anhui | B1 | - |
| 79 | DE058 | duck | Jiangsu | B1 | - |
| 80 | DE133 | duck | Anhui | B2 | - |
| 81 | DE184 | duck | Jiangsu | A | - |
| 82 | DE010B | duck | Jiangsu | B1 | - |
| 83 | DE020 | duck | Anhui | B1 | - |
| 84 | DE001B | duck | Jiangsu | A | - |
| 85 | DE021 | duck | Anhui | A | - |
| 86 | DE039 | duck | Anhui | D | - |
| 87 | DE050B | duck | Anhui | A | - |
| 88 | DE042 | duck | Anhui | A | - |
| 89 | DE143 | duck | Shandong | B1 | - |
| 90 | DE287 | duck | Jiangsu | A | - |
| 91 | DE449 | duck | Anhui | A | - |
| 92 | DE141 | duck | Shandong | B1 | - |
| 93 | DE429 | duck | Anhui | A | - |
| 94 | DE409 | duck | Jiangsu | D | - |
| 95 | DE109 | duck | Jiangsu | A | - |
| 96 | DE141 | duck | Shandong | B1 | - |
| 97 | DE457 | duck | Anhui | D | - |
| 98 | DE437 | duck | Anhui | A | - |
| 99 | DE459 | duck | Anhui | D | - |
| 100 | DE024 | duck | Anhui | A | - |
| 101 | DE111 | duck | Jiangsu | A | - |
| 102 | DE148 | duck | Shandong | A | - |
| 103 | DE199 | duck | Anhui | B1 | - |
| 104 | DE176 | duck | Jiangsu | D | - |
| 105 | DE181 | duck | Jiangsu | A | - |
| 106 | DE006 | duck | Jiangsu | A | - |
| 107 | DE174 | duck | Jiangsu | D | - |
| 108 | DE428 | duck | Anhui | A | - |
| 109 | DE446 | duck | Anhui | A | - |
| 110 | DE430 | duck | Anhui | A | - |
| 111 | DE461 | duck | Anhui | D | - |
| 112 | DE154 | duck | Anhui | B1 | - |
| 113 | DE136 | duck | Shandong | D | - |
| 114 | DE290 | duck | Jiangsu | A | - |
| 115 | DE179 | duck | Jiangsu | A | - |
| 116 | DE016 | duck | Jiangsu | A | - |
| 117 | DE440 | duck | Anhui | A | - |
| 118 | DE450 | duck | Anhui | A | - |
| 119 | DE451 | duck | Anhui | A | - |
| 120 | DE285 | duck | Jiangsu | A | - |
| 121 | DE431 | duck | Anhui | A | - |
| 122 | DE136 | duck | Shandong | D | - |
| 123 | DE421 | duck | Jiangsu | A | - |
| 124 | DE280 | duck | Jiangsu | A | - |
| 125 | DE137 | duck | Shandong | B1 | - |
| 126 | DE106 | duck | Jiangsu | A | - |
| 127 | DE420 | duck | Jiangsu | A | - |
| 128 | DE198 | duck | Anhui | B1 | - |
| 129 | DE115 | duck | Jiangsu | A | - |
| 130 | DE445 | duck | Anhui | A | - |
| 131 | DE463 | duck | Anhui | D | - |
| 132 | DE287 | duck | Jiangsu | A | - |
| 133 | DE438 | duck | Anhui | A | - |
| 134 | DE140 | duck | Shandong | B1 | - |
| 135 | DE141 | duck | Shandong | B1 | - |
| 136 | DE292 | duck | Jiangsu | A | - |
| 137 | DE004 | duck | Jiangsu | A | - |
| 138 | DE145 | duck | Shandong | B1 | - |
| 139 | DE138 | duck | Shandong | B1 | - |
| 140 | DE439 | duck | Anhui | A | - |
