# Supplementary figures and images for: Iron-regulated gene ireA in avian pathogenic Escherichia coli participates in adhesion and stress-resistance
Source: BMC Vet Res. 2016 Aug 17;12:167. doi: 10.1186/s12917-016-0800-y (PMC4988017; doi:10.1186/s12917-016-0800-y)

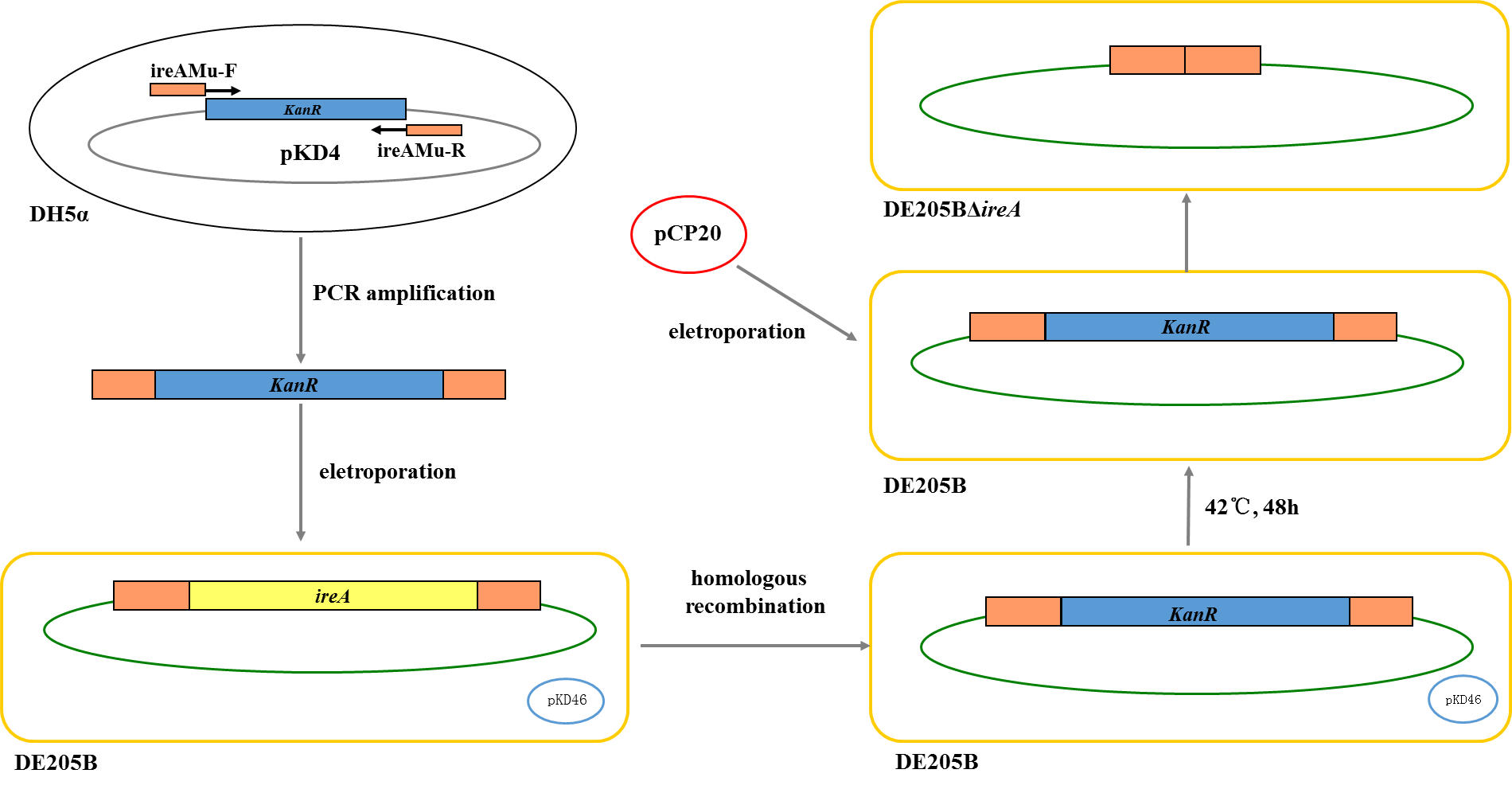

Supplement: Additional file 2: Figure S1. — Technical routes of construction of the ireA deletion mutant. (TIF 98 kb) [file 12917_2016_800_MOESM2_ESM.tif]

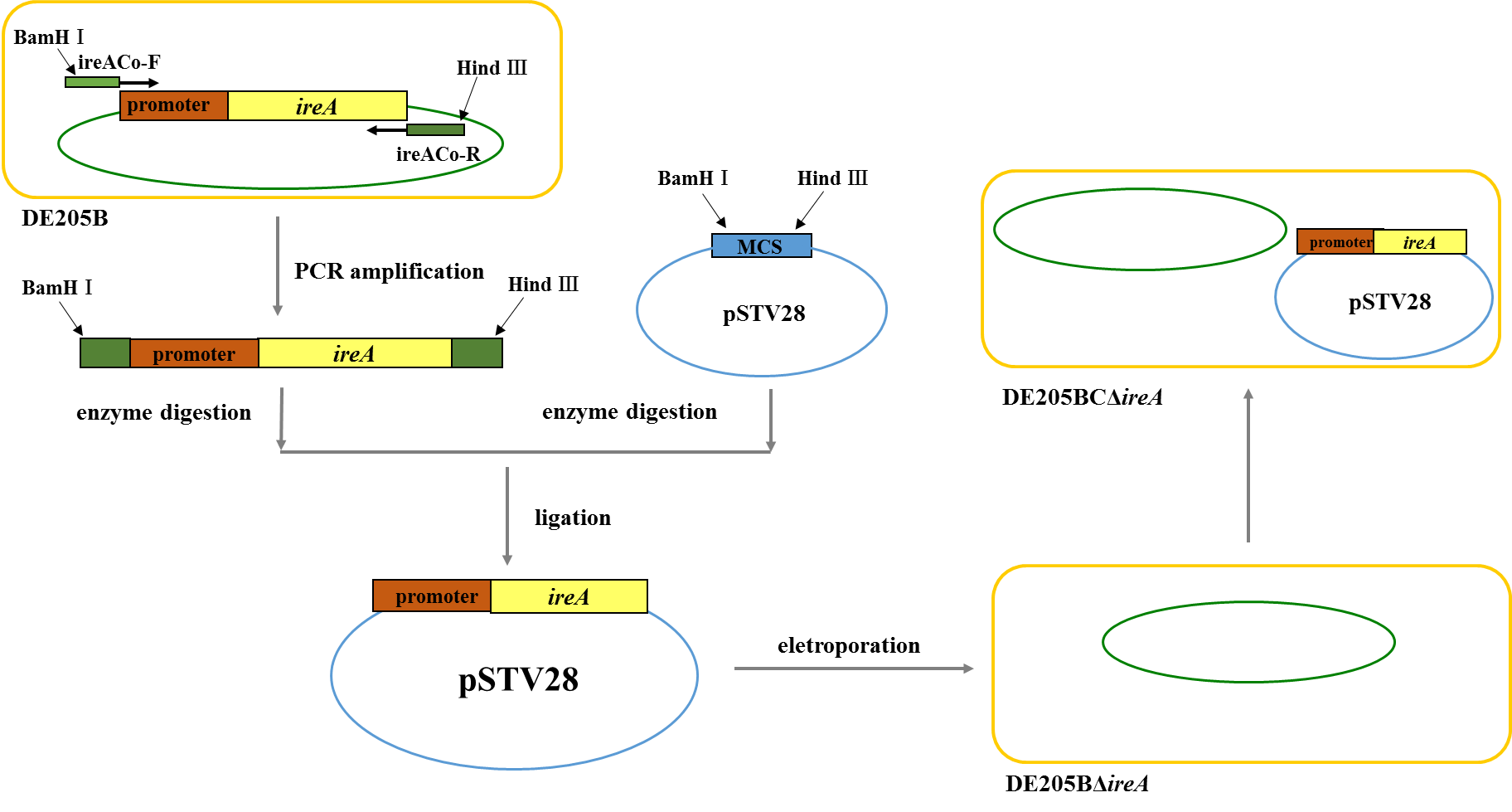

Supplement: Additional file 3: Figure S2. — Technical routes of construction of the ireA complementary strain. (TIF 102 kb) [file 12917_2016_800_MOESM3_ESM.tif]
